# Nuciferine Regulates Immune Function and Gut Microbiota in DSS-Induced Ulcerative Colitis

**DOI:** 10.3389/fvets.2022.939377

**Published:** 2022-07-13

**Authors:** Yiling Zhu, Qing Zhao, Qi Huang, Yana Li, Jie Yu, Rui Zhang, Jiali Liu, Pupu Yan, Jinjin Xia, Liwei Guo, Guoping Liu, Xiaolin Yang, Jianguo Zeng

**Affiliations:** ^1^College of Animal Science, Yangtze University, Jingzhou, China; ^2^College of Veterinary Medicine, Hunan Agricultural University, Changsha, China

**Keywords:** nuciferine, immune function, gut microbiota, ulcerative colitis, T cells, Th1/Th2 cells, Treg/Th17 cells

## Abstract

Nuciferine, a major aporphine alkaloid obtained from the leaves of *Nelumbo nucifera*, exhibits anti-cancer and anti-inflammatory properties; however, its protective effects against inflammatory bowel diseases (IBD) has never been explored. In this study, an ulcerative colitis (UC) model was established in BALb/c mice by the continuous administration of 5% dextran sulfate sodium (DSS) in drinking water for 1 week. From day 8 to day 14, the DSS-treated mice were divided into a high-dose and a low-dose nuciferine treatment group and were intraperitoneally injected with the corresponding dose of the drug. Body weight loss, disease activity index (DAI), and colon length were measured. Histological changes were observed using hematoxylin and eosin staining. T lymphocyte proliferation was assessed by MTT assay. The ratio of CD3+, CD4+, CD8+, Th1, Th2, Th17, and Treg cells were estimated by flow cytometry. Finally, 16S rRNA sequencing was performed to compare the composition and relative abundance of the gut microbiota among the different treatment groups. The results showed that nuciferine treatment led to a significant improvement in symptoms, such as histological injury and colon shortening in mice with DSS-induced UC. Nuciferine treatment improved the Th1/Th2 and Treg/Th17 balance in the DSS-induced IBD model, as well as the composition of the intestinal microflora. At the phylum level, compared with the control group, the abundance of *Firmicutes* and *Actinobacteriota* was decreased in the model group, whereas that of Bacteroidetes increased. Meanwhile, at the genus level, compared with the control group, the numbers of the genera Lachnospiraceae_*Clostridium, Bilophila* and *Halomonas* reduced in the model group, while those of *Bacteroides, Parabacteroides*, and *Paraprevotella* increased. Notably, nuciferine administration reversed this DSS-induced gut dysbiosis. These results indicated that nuciferine modulates gut microbiota homeostasis and immune function in mice with DSS-induced UC.

## Introduction

Inflammatory bowel disease (IBD), encompassing ulcerative colitis (UC) and Crohn's disease, represents a group of common, chronic, recurrent, and destructive gastrointestinal inflammatory diseases of unclear etiology (1, 2). Increasing evidence has implicated genetic predisposition, an overactive immune response, and gut dysbiosis in the pathogenesis of IBD. These factors interact with each other, making the etiology of UC more complicated (3, 4). Relatively few UC-specific drugs are currently available for the treatment of this condition. Drugs currently used to treat IBD include 5-aminosalicylic acid, glucocorticoids, immunosuppressants, and biological agents, among others; however, these drugs are associated with a variety of adverse reactions and the development of drug resistance.

At present, natural metabolites and phytochemicals derived from plants generally exhibit low toxicity and are increasingly evaluated for their pharmacological effects in the treatment of diarrheal diseases (5, 6). Consequently, the development of experimental therapies derived from a variety of substances from different sources, including synthetic methods and natural separations has become a research hotspot (7, 8). Fecal bacteria transplantation represents a novel method for treating IBD based on rebuilding normal intestinal microecology (9). Probiotics can relieve IBD by helping to restore the gut microbiota (10); in contrast, the use of antibiotics can lead to serious side effects and complications, such as increased incidence of malignant tumors or infectious diseases (11).

Traditional Chinese medicine (TCM) represents an effective auxiliary means for the treatment of a variety of diseases. With its multicomponent/multitarget characteristics, TCM has gradually become a key focus of clinical research in the treatment of intestinal diseases, especially UC-related cancer. Polysaccharides from *A. membranaceus* can reportedly attenuate colitis symptoms by inhibiting NF-κB (12). Studies have shown that 2,3,5,4′-tetrahydroxystilbene-2-O-β-D-glucoside, a major bioactive component derived from *radix polygoni multiflori* (Heshouwu), can suppress dextran sulfate sodium (DSS)-induced acute colitis in mice by modulating the gut microbiota (13). Similarly, *Camellia sinensis* and *Litsea coreana* were reported to ameliorate intestinal inflammation and modulate gut microbiota in mice with DSS-induced colitis (14). In addition, *Flos Abelmoschus manihot* extract was shown to attenuate DSS-induced colitis by modulating gut microbiota homeostasis and the T helper 17 (Th17)/regulatory T cell (Treg) balance (15). These studies in animals have suggested that the gut microbiota and Th17/Treg balance play a critical role in the development of IBD.

Lotus rhizome has been consumed as food in China for thousands of years. Lotus seeds, germs, and leaves are used in TCM to treat fever, diarrhea, and bleeding (16). Nuciferine, an aromatic ring-containing alkaloid, is a major bioactive component derived from lotus leaves (17). It has been shown to ameliorate high-fat diet-induced obesity in mice by regulating the intestinal microflora (18, 19), improve the fructose or uric acid-induced inflammation in HK-2 cells (20), mastitis in mice (21), and cerebral ischemia in rats (22). Despite its documented excellent anti-inflammatory activity, little is known about its potential pharmacological effects on IBD and its effect on Th17/Treg cell balance.

In the present study, we evaluated whether nuciferine exerted protective effects in mice with DSS-induced UC and whether nuciferine could reestablish CD4+/CD8+ and Th17/Treg cell homeostasis. We also investigated the effect of nuciferine on intestinal flora composition by 16S rRNA sequencing analysis.

## Materials and Methods

### Materials

Dextran Sulfate Sodium Salt (DSS) was purchased from Yeasen Biotech Co. Ltd (Shanghai, China). The molecular weight of DSS was 36,000~50,000. Nuciferine was purchased from Sichuan Vicky Biotechnology Co. Ltd (Chengdu, China). Its chemical formula was C19H21NO2, the molecular weight was 295.38, and the purity measured by HPLC was equal to or >98%. DMEM and FBS were purchased from Thermo Fisher Scientific (Shanghai, China). Red blood cell lysate, 4% paraformaldehyde, phytohemagglutinin (PHA), Methyl Thiazolyl Tetrazolium (MTT), dimethylsulfoxide (DMSO), penicillin and streptomycin were purchased from Solarbio Life Sciences Co. Ltd (Beijing, China). All monoclonal antibodies for flow cytometry detecting were purchased from BD Biosciences (New Jersey, USA). Antibodies of occludin, ZO-1, and claudin for immunohistochemistry detecting were purchased from Wuhan Servicebio Technology Co., Ltd (Wuhan, China). QIAamp Fast DNA Stool Mini Kit were purchased from Qiagen (Hilden, Germany). Urine fecal occult blood test kits were purchased from Shanghai Chemtron Biotech. Co. Ltd (Shanghai, China). Agencourt AMPure Beads were purchased from Beckman Coulter Co. Ltd (Indianapolis, USA). PicoGreen dsDNA Assay Kit was purchased from Invitrogen (California, USA).

### Methods

#### Animals

Six-week-old male BALb/c mice, weighing 20 ± 2 g, were purchased from the Center for Animal Experiments of Wuhan University (Hubei, China). They were housed under a specific pathogen-free environment under controlled conditions (12:12 h light-dark cycle, temperature of 25 ± 2°C, and relative humidity of 50 ± 5%) and had free access to standard laboratory diet and water during the experimental period. After a 1-week acclimatization period, the mice were grouped according to weight. All animal handling procedures strictly complied with the Legislation on the Use and Care of Laboratory Animals of the People's Republic of China and were approved by the Animal Care Review Committee of Yangtze University.

#### Experimental Design and Management

Forty male BALb/c mice were randomly divided into five groups as follows: blank control (untreated) group (Control); DSS-only group (DSS); DSS+low-dose [10 mg/(kg·day)] nuciferine group (L-Nuc+DSS); DSS+high-dose [20 mg/(kg·day)] nuciferine group (H-Nuc+DSS); and nuciferine-only control treatment group (Nuc). Mice in the DSS and DSS+Nuc groups were administered with 5% DSS in drinking water for 7 days. Mice in Control and Nuc groups were given regular drinking water without DSS. From Day 8 to Day 14, mice in H-Nuc+DSS, L-Nuc+DSS, and Nuc groups were administered with the corresponding dose of nuciferine by intraperitoneal injection. The disease activity index (DAI) scores were determined on the last day.

#### Sample Collection

On Day 15, all the mice were sacrificed by cervical dislocation. The spleen, mesenteric lymph nodes (MLN), and Peyer's patches (PP) were removed, sterilized with alcohol, and grounded in DMEM containing 10% FBS, 100 U/mL penicillin, and 100 μg/mL streptomycin. MLN and spleen tissue was grounded to isolate single cells and remove the red blood cells using red blood cell lysate. The cell suspension was then filtered through a 300-mesh membrane and centrifuged at 2,000 rpm for 5 min at 4°C. The pellet was washed with FACS buffer (PBS, 0.1% BSA) and the cells were counted. The colons were immediately and completely removed to determine the colon length. After washing with normal saline, a portion of each colon was fixed in 4% paraformaldehyde for histological analysis. Feces were collected and all samples were stored at −80°C for subsequent analysis.

#### Evaluation of DSS-Induced Colitis

The DAI was evaluated using the standard scoring system for weight loss, stool consistency, and bloody feces (23). Weight loss was calculated as the percentage difference between the original body weight (day 0) and the body weight on any day. Bleeding in feces was assessed using urine fecal occult blood test kits (0: normal; 1: blue-green in 30 s; 2: blue-green in 10 s; 3: blue-green immediately; 4: blue immediately). Stool consistency was scored as follows: 0, normal; 1, soft but still formed; 2, very soft; 3, half diarrhea; 4, diarrhea. The colon tissues were fixed in 4% paraformaldehyde for 48 h, embedded in paraffin, cut into 4-μm-thick slices, hydrated, subjected to hematoxylin and eosin (H&E) staining, and finally observed under a microscope.

#### Quantification of Occludin, Zona Occludens 1 (ZO-1), and Claudin Levels in the Colon by Immunohistochemistry

Distal colon tissues were fixed in 4% paraformaldehyde for 24 h, embedded in paraffin, and cut into 5-μm-thick sections. The tissues were subsequently deparaffinized, rehydrated, and incubated with primary antibodies against occludin, ZO-1, and claudin at 37°C. The sections were stained with DAB, counterstained with hematoxylin, and observed under a Leica microscope. Five fields were observed for each section. Images were processed using ImageJ software.

#### Activation of B220 Cells and B220-IgA

PP node cells (2.5 × 106 cells/mL) were stained with an APC anti-mouse CD45R/B220 monoclonal antibody and a FITC anti-mouse IgA antibody (BD Biosciences, USA) and detected by flow cytometry. Flow cytometry data were analyzed using FlowJo v10 software.

#### T-Cell Proliferation in the Spleen and Lymph Nodes

Cell proliferation was assessed by MTT assay. Spleen and mesenteric lymph node cells (2.5 × 10^6^ cells/mL) were seeded in 96-well plates, 80 μL per well. Then, 20 μL of 50 mg/mL phytohemagglutinin (Solarbio) was added to each well and the cells were incubated at 37°C for 48 h in a humidified atmosphere with 5% CO_2_. After 44 h of incubation, 20 μL of MTT (5 mg/mL) was added to each well. After incubation for 4 h, the culture medium was removed and 150 μL of DMSO was added. The plates were shaken for 5 min to completely dissolve the formazan crystals. The absorbance at 570 nm (*A*_570_ value) was measured using an enzyme-linked immunosorbent assay microplate reader.

#### Detection of T Cell Subtypes by Flow Cytometry

MLN cells (1 × 10^6^ cells/mL) were stained with PE anti-mouse CD8a, Percpcy5.5 anti-mouse CD4, and FITC anti-mouse CD3e monoclonal antibodies (10 μL) (BD Biosciences) for 15 min at room temperature in the dark. Stained cells were washed twice with PBS and fixed in 300 μL of PBS. Spleen cells (1 × 10^6^ cells/mL) were stained with FITC anti-mouse CD4, APC anti-mouse IL-17a, PE anti-mouse IFN-γ, Percpcy5.5 anti-mouse IL-4, Percpcy5.5 anti-mouse CD4, PE anti-mouse CD25, and APC anti-mouse FOXP3 monoclonal antibodies. Flow cytometry data were analyzed using Flow Jo v10 software.

#### DNA Extraction From Mouse Feces and 16S RRNA Gene Amplification

Microbial community composition in mouse feces was analyzed by high-throughput sequencing of the 16S rRNA gene (Shanghai Personal Biotechnology Co., Ltd, China). Total DNA was extracted from intestinal bacteria using the QIAamp Fast DNA Stool Mini Kit according to the manufacturer's protocol (24). The V3 and V4 hypervariable regions of the bacterial 16 S rRNA were amplified from extracted fecal DNA using barcoded primers (13). The primer sequences were as follows: 338 F (5′-ACTCCTACGGGAGGCAGCAG-3′) and 806 R (5′-GGACTACHVGGGTWTCT AAT-3′) (25). The PCR products were detected by electrophoresis and purified using Agencourt AMPure Beads. The purified samples were used as templates for two-round PCR. The resulting products were again detected by electrophoresis, purified using Agencourt AMPure Beads, and quantified using the PicoGreen dsDNA Assay Kit. According to the sample sequencing requirements, the corresponding proportion of the mix. Sequencing (2 × 300 bp) sequencing was performed using the Illumina MiSeq platform with MiSeq Reagent Kit v3 at Shanghai Personal Biotechnology Co., Ltd, China. The raw FASTQ files were analyzed using QIIME platform scripts and the microbial classification was performed with the GreenGenes reference database using QIIME tools. Alpha and beta diversity were analyzed for each library using QIIME. Moreover, observed species index and the principal component analysis.

### Statistical Analysis

SPSS v.25.0 (SPSS, Chicago, IL, USA) and GraphPad Prism v.8.0.2 (GraphPad Software, San Diego, CA, USA) were used to analyze the data and construct the graphs. Data are presented as means ± standard deviation (SD). Significant differences among groups were detected using one-way ANOVA followed by Tukey's multiple-comparison test. A *p* < 0.05 was considered statistically significant.

## Results

### Nuciferine Ameliorated the Symptoms of DSS-Induced Colitis

**Figure 1:**
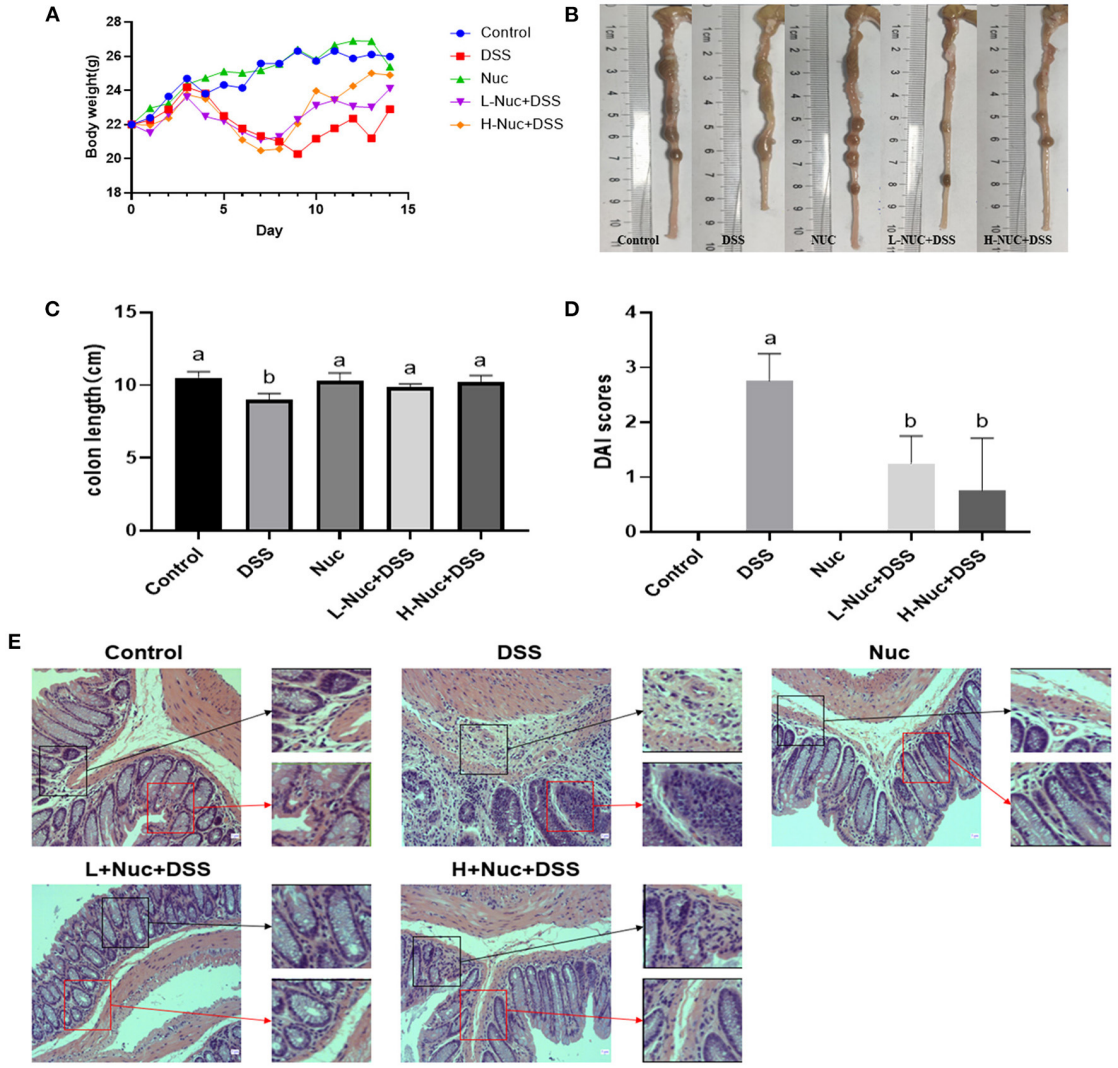
Nuciferine reduced clinical signs in DSS-activated colitis (*n* = 6). **(A)** The illustrative images of colon samples **(B)** Average body weight **(C)** Average colon length and **(D)** Disease activity index score. **(E)** H&E staining of the distal colon tissues The dataare expressed as the mean ± S.D. Significant differences were considered at *P* < 0.05. ^a,b^ Bars without the same superscripts differ significantly (*P* < 0.05).

Mice with DSS-induced colitis (5% DSS administered in drinking water for 7 days) showed a significant decrease in body weight from day 3 (Figure 1C). After 8 days of treatment, mice from both the low-dose and high-dose nuciferine groups had gained weight. Additionally, the colons of mice in the DSS-only treatment group were shorter than those of mice in the control group (Figures 1A,B); however, this phenotype was partially rescued following nuciferine treatment (high-dose and low-dose nuciferine). Notably, mice in both the high-dose and low-dose nuciferine treatment groups displayed better DAI scores than mice in the DSS treatment group (Figure 1D). These results are similar to those of He et al. (13), who showed that DSS induces pathological changes in UC at both the macrocosmic and microcosmic levels. Pathological changes in mice from the DSS treatment group included the disruption of epithelial integrity and severely damaged colonic epithelial cells. Nuciferine treatment rescued epithelial layer rupture, a reduction in goblet cell numbers, and inflammatory cell infiltration resulting from DSS treatment (Figure 1E). These results showed that nuciferine effectively ameliorated DSS-induced symptoms in colitic mice.

### Nuciferine Regulated the Differentiation of B220+ and IgA+ Cells

**Figure 2:**
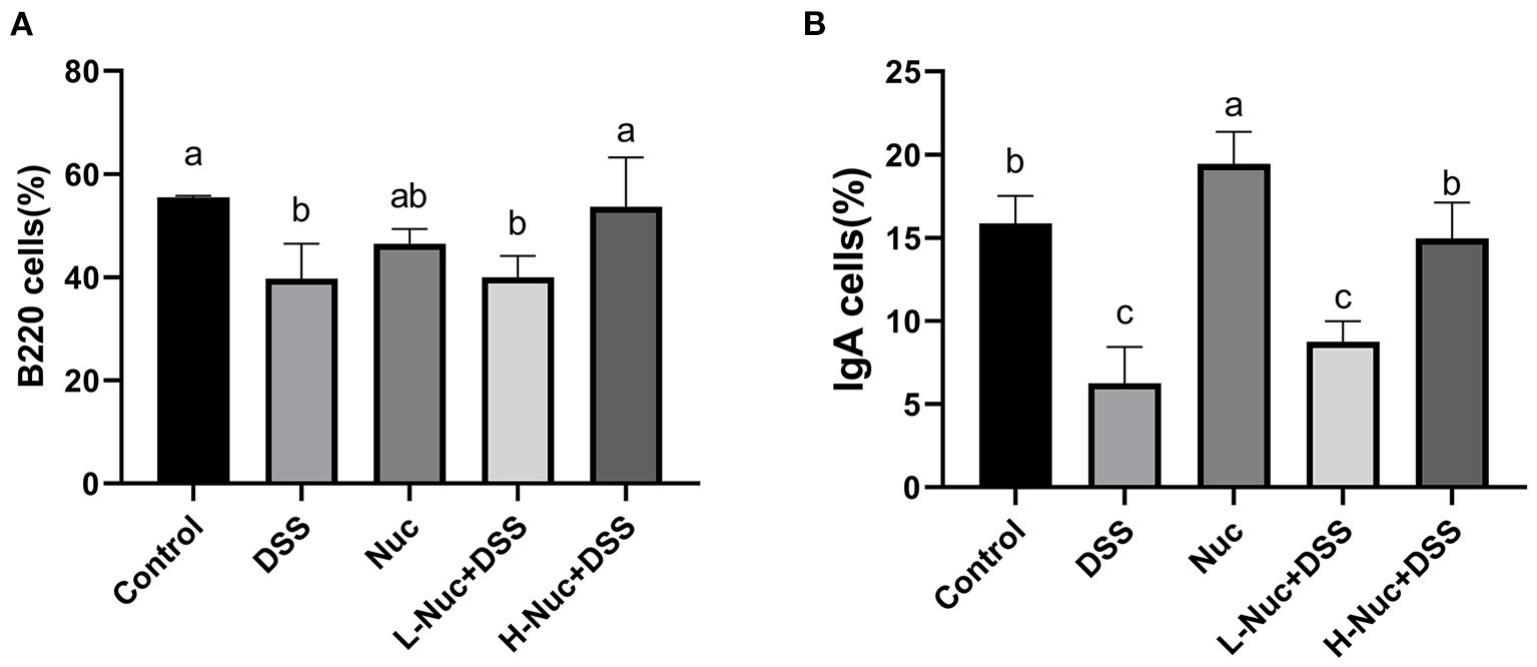
Effects of nuciferine on B cell, **(A)** The percentage of B220 cells in the PP nodes. **(B)** The percentage of IgA cells in the PP nodes (*n* = 6). The dataare expressed as the mean ± S.D. Significant differences were considered at *P* < 0.05. ^a−*c*^ Bars without the same superscripts differ significantly (*P* < 0.05).

To further evaluate the effect of nuciferine in mice with DSS-induced colitis, we next measured the levels of B220+ and IgA+ B cells in PP nodes by flow cytometry. As shown in Figure 2, compared with the DSS group, the levels of B220+ and IgA+ B cells in the PP nodes of mice in the high-dose treatment group were significantly increased (*P* < 0.05), while the levels of IgA+ B cells in the low-dose group also showed an upward trend. In contrast, compared with the control group, the levels of B220+ and IgA+ B cells in the DSS group were significantly decreased (*P* < 0.05). These results showed that nuciferine exerted a regulatory effect on the differentiation of both B220+ and IgA+ cells.

### Nuciferine Promotes T-Cell Proliferation and Differentiation

**Figure 3:**
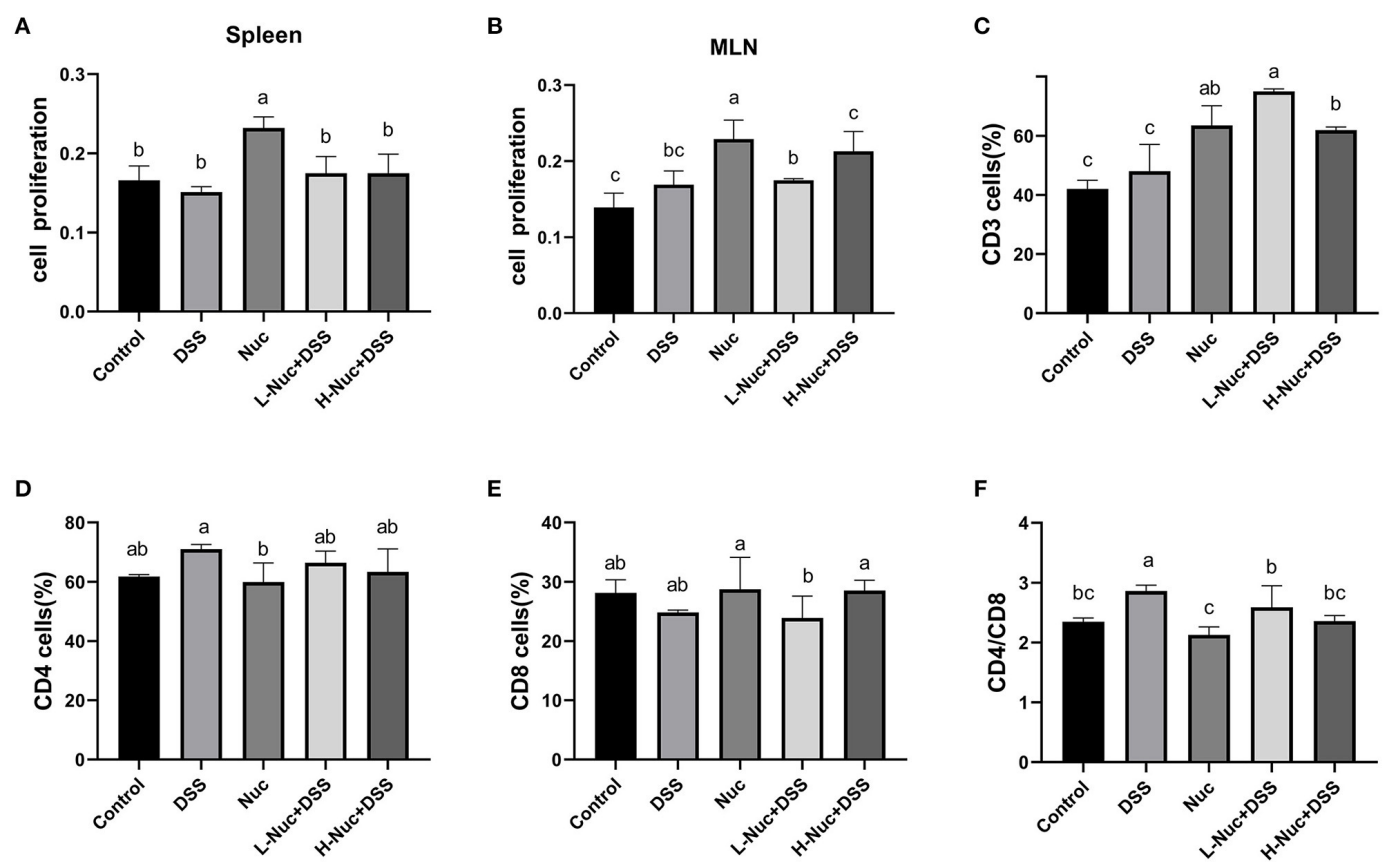
Nuciferine regulated T cell proliferation and differentiation (*n* = 6). Effects of nuciferine on T cell proliferation in spleen **(A)** and mesenteric lymph nodes **(B)**. **(C)** The percentage of CD3 cells in the mesenteric lymph nodes. **(D)** The percentage of CD4 cells in the mesenteric lymph nodes. **(E)** The percentage of CD8 cells in the mesenteric lymph nodes. **(F)** Comparison on the ratio of CD4/ CD8 between groups, The dataare expressed as the mean ± S.D. Significant differences were considered at *P* < 0.05. ^a−*c*^ Bars without the same superscripts differ significantly (*P* < 0.05).

The MTT assay was used to detect the effect of nuciferine on T-cell proliferation in the spleen and mesenteric lymph nodes. As shown in Figure 3, T-cell proliferation levels were significantly higher in the spleen and mesenteric lymph nodes of the high-dose and low-dose nuciferine treatment groups relative to control mice (*P* < 0.05), while T-cell proliferation levels in the nuciferine treatment groups were higher than those in the DSS group (Figures 3A,B). These results indicated that nuciferine treatment can significantly increase T-lymphocyte proliferation and enhance cellular immune responses.

The levels of CD3+, CD4+, and CD8+ cells in mice with DSS-induced colitis were detected by flow cytometry, as shown in Figures 3C–F. Compared with DSS-only treatment, the number of CD3+ cells was significantly increased after treatment with nuciferine (*P* < 0.05). Compared with the control group, no significant difference between the numbers of CD4+ and CD8+ cells were observed in the DSS group; however, the CD4+/CD8+ cell ratio was significantly increased in the DSS group (*P* < 0.05). No significant differences were found between the nuciferine treatment groups and the control group. These results suggested that nuciferine plays a vital role in maintaining the homeostasis of a variety of T cell subtypes in the colons of mice with IBD.

### Nuciferine Restored the Th1/Th2 and Th17/Treg Balance

#### The Balance of Th1/Th2 Was Restored by Treatment of Nuciferine in Mice

**Figure 4:**
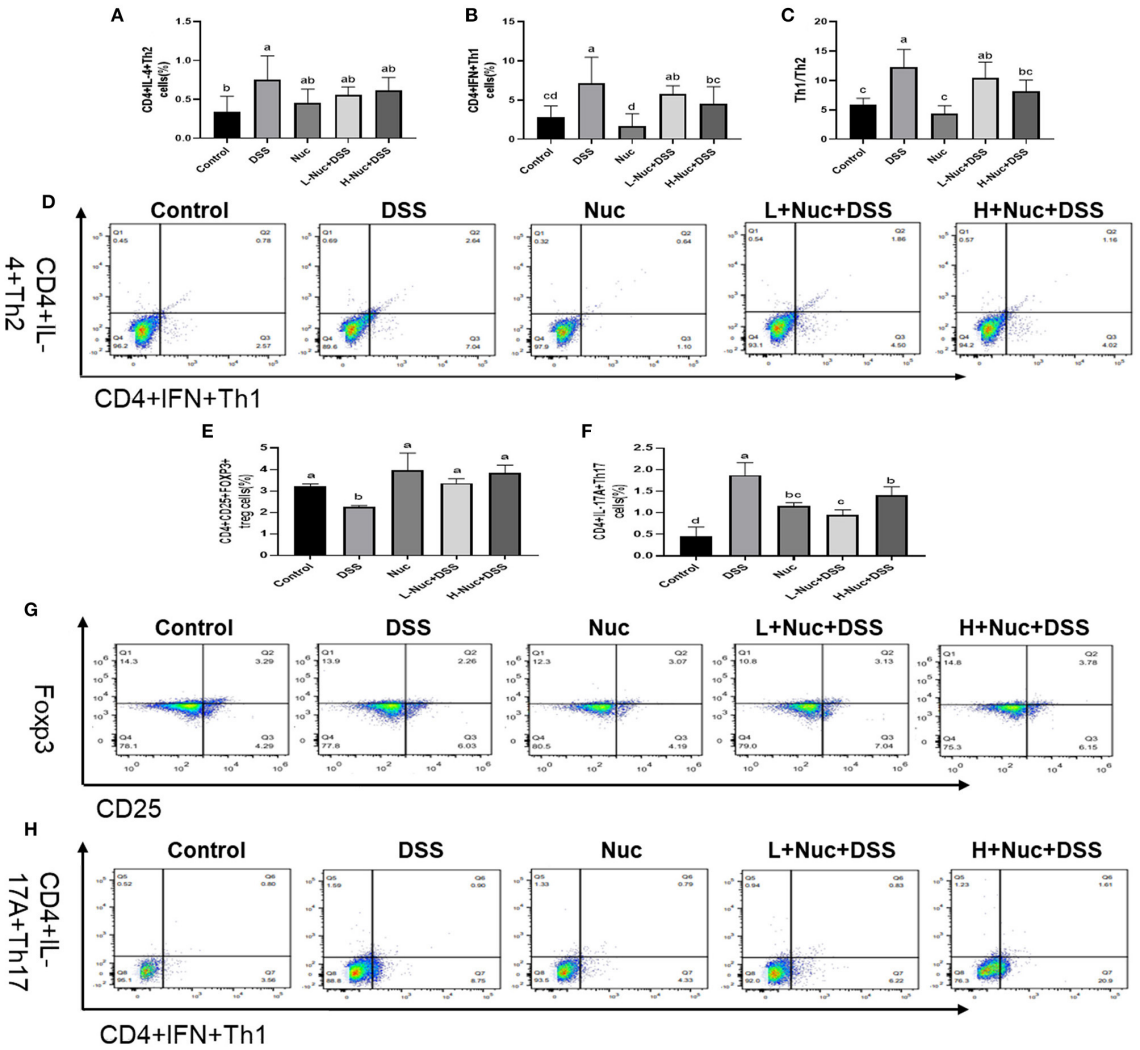
Nuciferine restored the balance of Th1/Th2 Th17/Treg cells (*n* = 6). **(A)** The percentage of IL-4 cells in the spleens. **(B)** The percentage of IFN cells in the spleens. **(C)** The Th1/Th2 ratio in the spleens. **(D)** Flow cytometry of IL-4 and IFN in the spleens. **(E)** The percentage of Tregs cells in the spleens. **(F)** The percentage of Th17 cells in the spleens. **(G)** Flow cytometry of Tregs in the spleens. **(H)** Flow cytometry of Th17 in the spleens. The data are expressed as the mean ± S.D. Significant differences were considered at *P* < 0.05. ^a−*d*^ Bars without the same superscripts differ significantly (*P* < 0.05).

To further assess the effect of nuciferine in DSS-induced colitis, the proportions of Th1 and Th2 cells in the spleen were measured by flow cytometry. As shown in Figures 4A–D, compared with the control group, the frequency of CD4+IFN-γ+ (Th1) and CD4+IL-4+ (Th2) cell subsets was significantly increased in the spleens of mice in the DSS group (*P*<0.05); however, nuciferine treatment significantly attenuated this DSS-induced effect in both cell subset types (both *P* < 0.05). Compared with the control group, the Th1/Th2 ratio was significantly increased in the DSS group (*P* < 0.05), whereas the opposite was seen with nuciferine treatment. These results demonstrated that DSS treatment promoted an imbalance in the Th1/Th2 ratio, and that nuciferine could restore this balance, at least to some extent.

#### The Balance of Th17/Treg Was Restored by Treatment of Nuciferine in Mice

As shown in Figure 4, the proportion of IL-17A-expressing cells was markedly enhanced in the spleens of DSS-treated animals compared with that in the control group (*P* < 0.05) (Figure 4F); however, nuciferine treatment reversed this trend. The proportion of Th17 cells in the nuciferine treatment groups was significantly lower than that in the DSS group. The percentage of Tregs in the spleens of the DSS group was noticeably reduced when compared with that in the spleens of mice in the nuciferine treatment groups (*P* < 0.05) (Figure 4E). Compared with the control group, the proportion of Tregs in the spleens of mice in the nuciferine treatment group was significantly lower than that in the spleens of mice in the control group (*P* < 0.05) (Figure 4E). These results indicated that nuciferine treatment restored the Th17 cell/Treg balance likely through regulating the differentiation and function of both cell types.

### Nuciferine Altered Gut Microbiota in DSS Induced Ulcerative Colitis

#### Analysis of the Diversity of the Gut Microbiota

**Figure 5:**
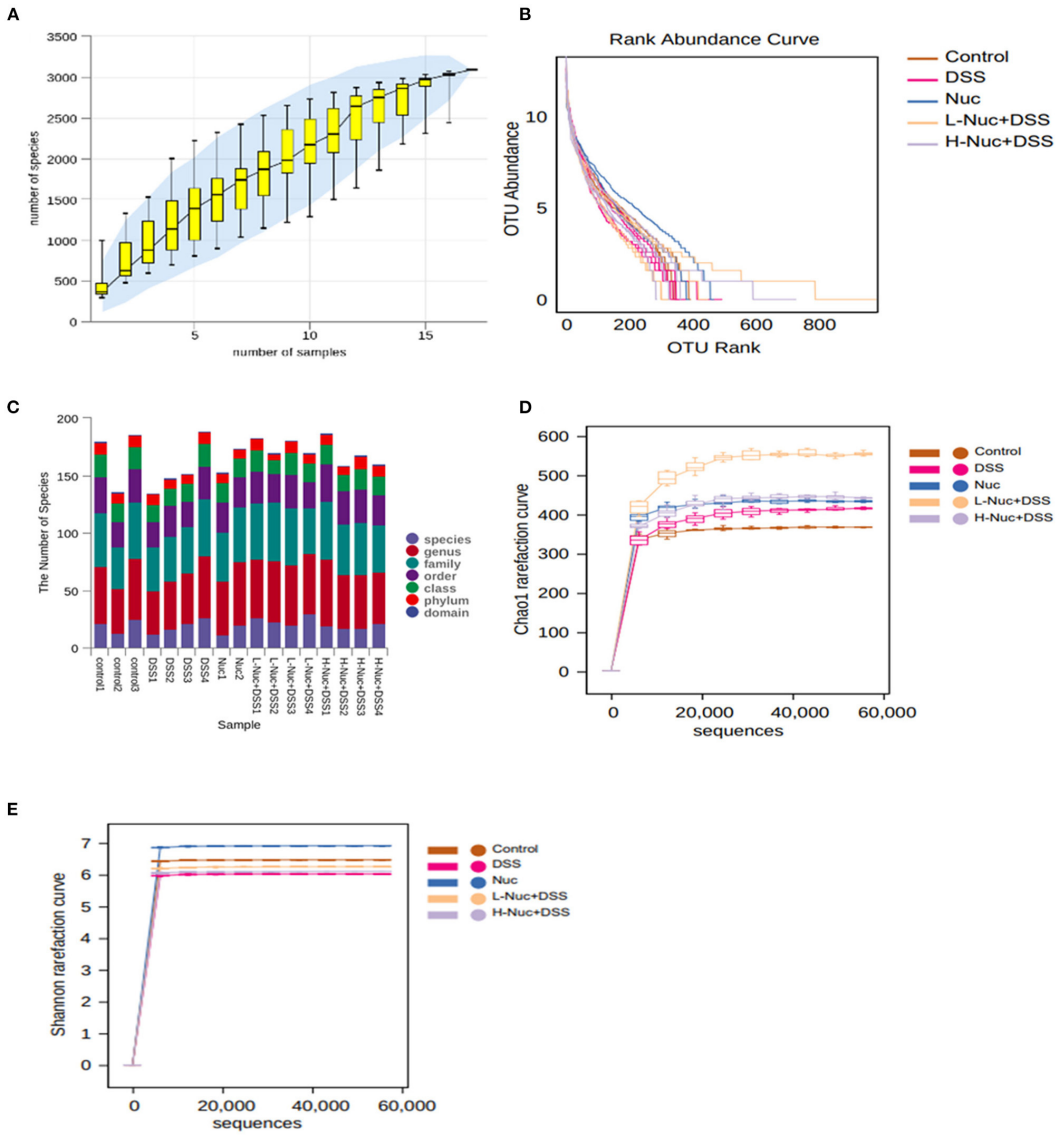
Species annotation and evaluation (*n* = 4). **(A)** pecies accumulation curve of samples. **(B)** Operational taxonomic units (OTU) relative abundance in individual samples of the control, DSS, Nuc, L-Nuc DSS and H-Nuc DSS groups. **(C)** Statistical chart of microbial groups at each classification level. The abscissa is arranged according to the sample name, and the abscissa is the number of microbial groups contained in each of the six classification levels of phylum, class, order, family, genus, and species. **(D)** Chao1 rarefaction curve. **(E)** Shannon rarefaction curve.

The relative species abundance and diversity of the gut microbiota in the nuciferine treatment groups were significantly greater than those of the DSS group (Figures 5A–E). A species accumulation curve was drawn (using R software) for the total number of operational taxonomic units (OTUs) corresponding to each sample in the OTU abundance matrix (Figure 5A). The rank-abundance curve, which evaluates species abundance and evenness, further confirmed that the sample size used for sequencing in this study was sufficient to satisfy the data analyses. Additionally, the species representing the microbial environment were evenly distributed and displayed wide range coverage (Figure 5B). The curve tended to flatten with increasing sample size, indicating that the sample size of this experiment was enough to reflect the richness of the community. The chao1 (Figure 5C) and Shannon (Figure 5D) rarefaction curves of all the groups tended to be stable with increasing numbers of gene sequences, which validated the reliability of the sequencing data. All the rarefaction curve results and the Shannon curve index indicated that sequencing coverage was sufficient for further data analysis.

#### Analysis of the Composition of Intestinal Flora in Different Treatment Groups

**Figure 6:**
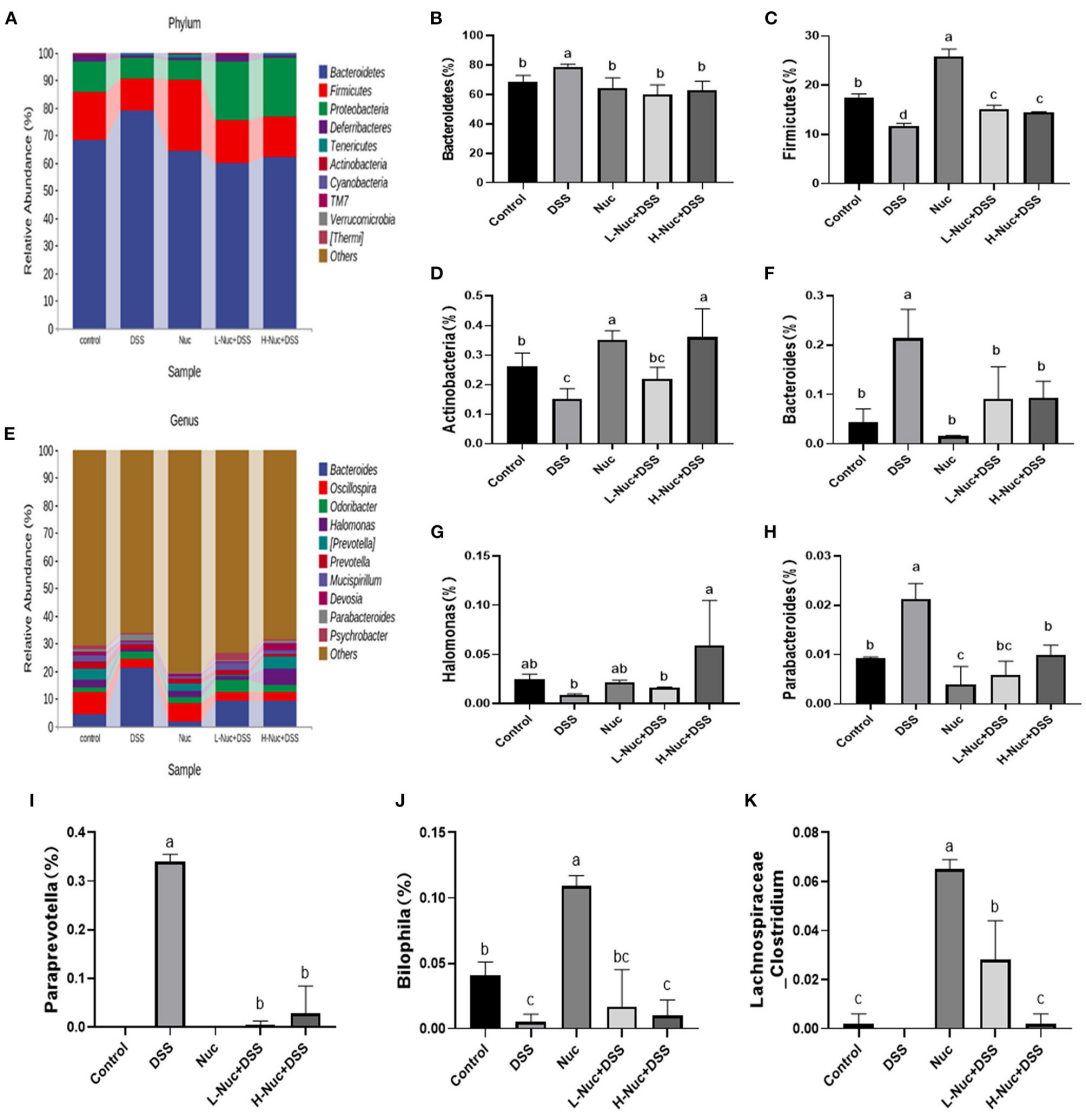
Change of gut microbiota composition in DSS-induced UC mice model under different drug interventions. **(A)** Gut microbial compositions at phylum level between groups, **(B,C)** Comparison on relative abundances of Bacteroidetes between groups, **(C)** Comparison on relative abundances of Firmicutes between groups **(D)**, Comparison on relative abundances of Actinobacteria between groups, **(E)** Gut microbial compositions at genus level, **(F)** Comparison on relative abundances of Bacteroides between groups **(G)** Comparison on relative abundances of Halomonas between groups, **(H)** Comparison on relative abundances of Parabacteroides between groups, **(I)** Comparison on relative abundances of BacteroideParaprevotellas between groups, **(J)** Comparison on relative abundances of Bilophila between groups. **(K)** Comparison on relative abundances of Lachnospiraceae_Clostridium between groups The data are expressed as the mean ± S.D (*n* = 4). Significant differences were considered at *P* < 0.05. ^a−d^ Bars without the same superscripts differ significantly (*P* < 0.05).

In the present study, we also evaluated the effect of nuciferine on microbiome composition remodeling and examined the changes in bacterial abundance at the phylum and genus levels between the treatment groups. As shown in the heatmaps in Figure 6A, at the phylum level, Firmicutes and Bacteroidetes were the most important classification in all the samples (26), accounting for 75.6–91.6% of the total abundance. Compared with the control group, mice in the DSS treatment group had a lower abundance of Firmicutes and Actinobacteriota, but a greater abundance of Bacteroidetes (Figures 6B–D). The above data suggested that gut microbiota homeostasis was disturbed in mice with DSS-induced UC. In contrast, nuciferine treatment increased the abundance of Firmicutes relative to the DSS treatment group (*P* < 0.05) (Figure 6C). Additionally, nuciferine treatment had a better effect on increasing the relative of Firmicutes. These results suggested that nuciferine could help restore the balance in the intestinal flora of mice with UC. There were obvious differences in the abundance of 50 genera between the control and the DSS groups (Figure 6E). The abundance of Lachnospiraceae_ *Clostridium, Bilophila*, and *Halomonas* in the DSS group was lower than that in the control group (*P* < 0.05), whereas that of *Bacteroides, Parabacteroides, Paraprevotella* was higher (*P* < 0.05) (Figures 6F–K). After nuciferine administration, the abundance of Lachnospiraceae _*Clostridium, Bilophila*, and *Halomonas* was significantly enhanced compared with DSS treatment (Figures 6G,J,K). These results suggested that nuciferine can help restore the balance in the intestinal flora in mice with DSS-induced UC.

#### Changes in the Structure of the Intestinal Microbiota

**Figure 7:**
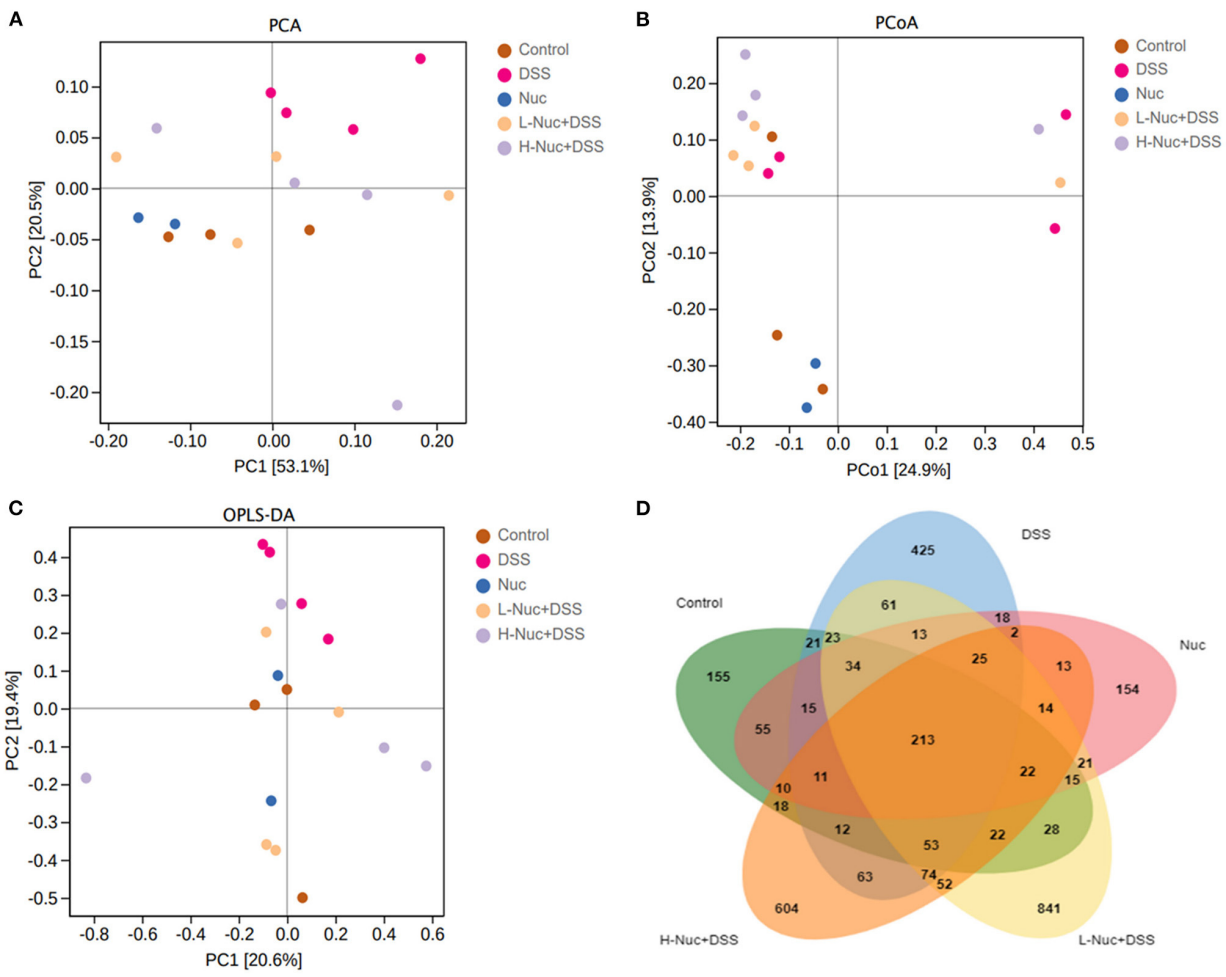
Change of gut microbiota structure in DSS-induced UC mice model under different drug interventions (*n* = 4). **(A)** Principal components analysis (PCA) for DSS- induced UC mice model with different treatments. **(B)** Principal coordinates analysis (PCoA) for DSS-induced UC mice model with different treatments. **(C)** Orthogonal Partial Least Squares Discriminant Analysis, and **(D)** Venn diagram analysis.

The results of the PCA showed that the control and DSS groups were distributed in different quadrants in the PCA plot, indicating that DSS administration had altered the structure of the gut microbiota (Figure 7A). Moreover, the Nuc, H-Nuc+DSS and L-Nuc+DSS groups were distributed very close to the control group, indicating that nuciferine administration might contribute to the remodeling of the structure of the microbiota in colitic mice (Figure 7A). Principal coordinates analysis (PCoA) based on Bray_curtis distance at the OTU level showed an obvious separation among the control, DSS, and nuciferine treatment groups. The first and second principal coordinates accounted for 24.9 and 13.9% of the variance, respectively (Figure 7B). Next, we used orthogonal partial least squares discriminant analysis (OPLS-DA) to explore the differences in the composition of the intestinal microflora among the different groups. The results showed that the distance between the two nuciferine treatment groups (low dose and high dose) and the control group was closer than that between the UC group, which indicated that nuciferine administration could reduce the influence of carcinogenic factors on the composition of intestinal flora (Figure 7C). Venn diagram analysis showed that 213 OTUs were shared among the five treatment groups. In total, 155 and 425 OTUs were unique to the control and DSS groups, respectively, while 841 and 604 OTUs were unique to the low-dose and high-dose nuciferine groups, respectively (Figure 7D). These results demonstrated that nuciferine could modulate the gut microbiota in mice and promote a high diversity of the intestinal microbiota.

### Nuciferine Treatment Ameliorated Intestinal Mucosal Damage

**Figure 8:**
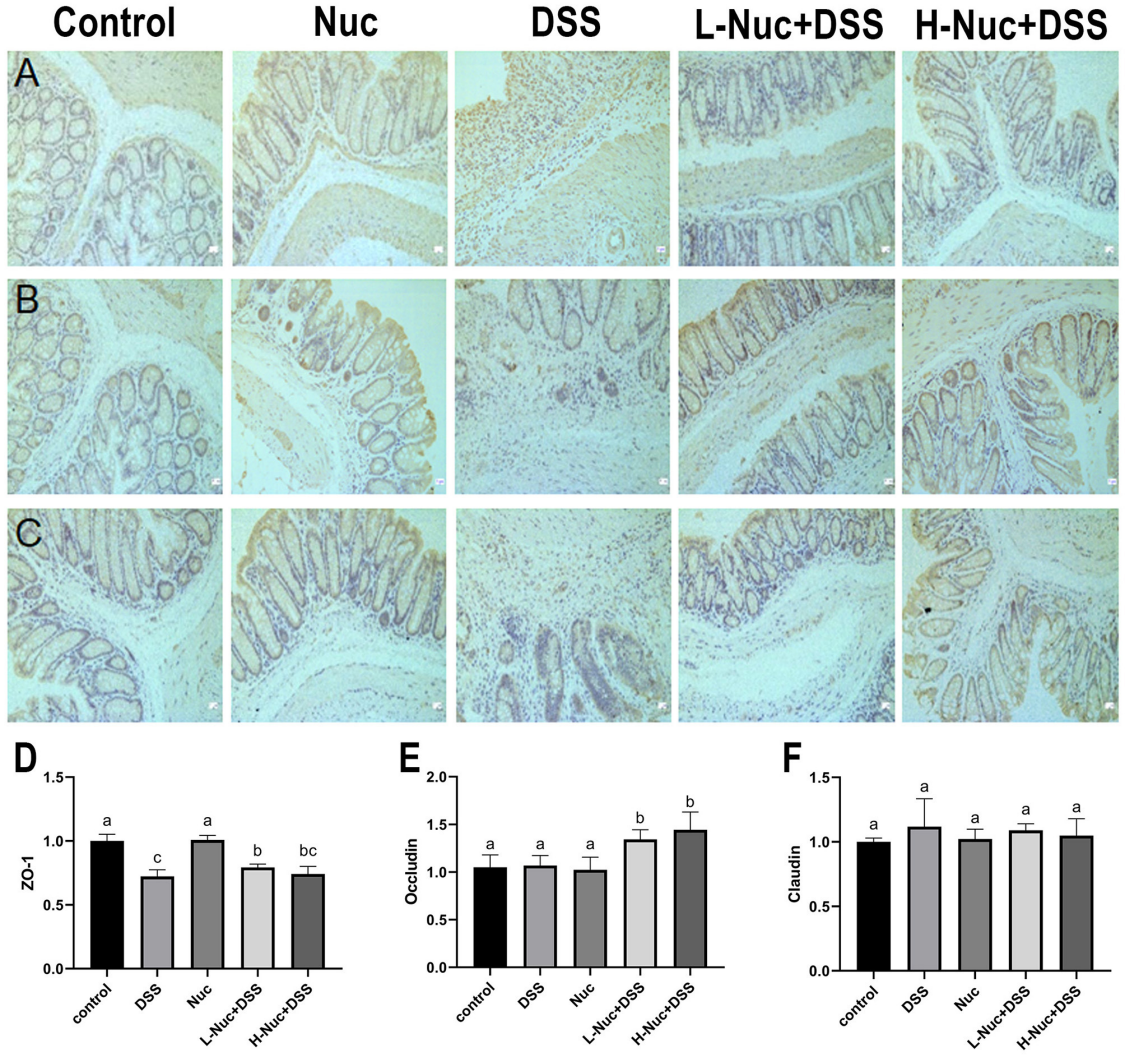
Nuciferine repaired intestinal mucosal damage. **(A)** Representative immunohisto chemical (IHC) staining of ZO-1. **(B)** IHC staining of occludin. **(C)** IHC analysis of claudin-1 in colon tissues. **(D)** Immunohistochemical analysis of ZO-1. **(E)** Immunohistochemical analysis of occludin. **(F)** Immunohistochemical analysis of claudin-1. The dataare expressed as the mean ± S.D (*n* = 6). Significant differences were considered at *P* < 0.05. ^a−*c*^ Bars without the same superscripts differ significantly (*P* < 0.05).

In this study, the results obtained by immunohistochemical staining of colon tissues showed in Figure 8. The expression of ZO-1 (Figure 8D) in the DSS group was markedly lower than that in the control group, while there were increasing expression of these substances witnessed in the H-Nuc+DSS and L-Nuc+DSS groups. In addition, the expression of occludin in the H-Nuc+DSS and L-Nuc+DSS groups was significantly upregulated relative to that in the DSS group (Figure 8E). No significant differences in claudin expression were found among the groups (Figure 8F). The results illustrated that nuciferine exerted protective effects on the intestinal barrier and relieved the symptoms of colitis.

## Discussion

Nuciferine is an aporphine alkaloid derived from the dried leaves of *Nelumbo nucifera* and is one of the quality control components for Chinese traditional medicine compiled by the Chinese pharmacopeia. Recent studies have shown that nuciferine can help reduce fat and weight; protect the liver (27, 28), kidney (29), and the nervous system; regulate glycometabolism (30); and exert anti-tumor (31), anti-inflammatory, and anti-oxidative effects (32). Nuciferine has also been shown to inhibit the proliferation of mouse CT26 colon cancer cells (33). In addition, nuciferine derived from lotus species was reported to modulate the abundance and diversity of intestinal flora and reduce intestinal permeability (22, 27). However, whether nuciferine has similar protective and ameliorative effects on IBD remains unknown. To test this, in this study, we established a DSS-induced mouse model of UC, and found that nuciferine can improve disease symptoms, pathological characteristics, and the composition of the intestinal microflora; regulate the differentiation of T cells and IgA+ B220+ B cells; and restore the Th17/Treg and CD4+/CD8+ balance.

MyD88 expressed by B cells has been shown to inhibit DSS-induced exacerbation of inflammatory bowel disease by secreting IgA and IgM in response to damaging intestinal microbiota dissemination (34). IgA, a component of mucus, is the first line of defense (35) against the entry of intestinal bacteria into the host (36), Paes'knot is the origin of Liga secretion by B cells, and lamina propria is the site of action (37) in mucosal immunity, lGA played a crucial role (38). In this study, the numbers of IgA+ and B220+ cells decreased after DSS administration, but increased in the nuciferine treatment groups, indicating that nuciferine could enhance the immune capacity of B cells. Lymphocytes comprise the most important group of immune cells in the body and are the main mediators of the cellular immune response. CD3+, CD4+, and CD8+ T cells are the primary T cell subtypes. Compared with the control group, the numbers of CD4+ and CD4+/CD8+ T cells were significantly higher in the DSS group, whereas those of CD8+ cells were lower. The cells with abnormal T lymphocyte and subpopulations had immune disorders. However, T cell subsets and CD4+/CD8+ were recovered after treatment with lotus leaf alkaloid. Th1 cells are mainly involved in cellular immune responses, whereas Th2 cells are mainly involved in promoting humoral immunity (39). When the Th1/Th2 ratio is in dynamic equilibrium in the body, the immune system is in relative balance. An imbalance in this ratio has long been associated with IBD (40). Here, we found that DSS administration disrupted this balance to some extent; however, this effect could be partially reversed by nuciferine treatment.

Innate immune cells initiate an effective inflammatory response to external stimuli by secreting cytokines and eventually activating T cells and inducing an acquired immune response (41). CD4+ T cells can be directly induced into various types of T cells, including Th1, Th2, Th17, and Treg cells (42). During UC development, a high abundance of Th17 cells and a lack of Treg cells leads to inflammation of the gut (43). Accordingly, an imbalance between Tregs and Th17 cells is considered a key factor in UC pathogenesis, suggesting that the restoration of Th17/Treg homeostasis may be an effective strategy for the treatment of this condition. For instance, paeoniflorin can ameliorate the symptoms of UC by regulating dendritic cell-mediated Th17/Treg homeostasis (44); juglone modulates gut microbiota and the Th17/Treg balance in DSS-induced UC (45); and gegen qinlian decoction can relieve DSS-induced UC in mice by modulating Th17/Treg cell homeostasis *via* suppressing IL-6/JAK2/STAT3 signaling (46). Here, we showed that nuciferine also relieves colitis by regulating the Treg/Th17 cell balance. We found that during IBD development, an overabundance of Th17 cells and a deficiency of Tregs resulted in gut inflammation, symptoms that were alleviated by nuciferine treatment. These results indicate that nuciferine can restore Th17/Treg homeostasis, resulting in an improvement in the inflammatory environment in the bowel and, consequently, a relief of the symptoms of IBD. These considerations prompted us to explore the effect of nuciferine on the stability of the intestinal environment related to Th17/Treg homeostasis in IBD mice.

Studies have consistently shown that UC is caused by an overactive immune response in the gut, leading to inflammation and pathological damage (47). It is widely believed that UC results from an abnormal T-cell immune response to gut bacteria (48). Cooperation among different CD4+ T-cell subtypes contributes to the stability of the intestinal environment. Gut microbiota play a critical role in the development of IBD. It has been demonstrated that the intestinal flora is involved in the development of UC and affects its severity. In particular, intestinal flora dysbiosis promotes intestinal inflammation and an immune response (49), which may be key for the induction of IBD (50). When UC occurs, the intestinal flora becomes disordered, the abundance of harmful bacteria increases, and the number of beneficial bacteria decreases (51–53). Thus, the intestinal microflora may represent a promising therapeutic target for the treatment of IBD (54).

In this study, at the phylum level, the abundance of Firmicutes and Actinobacteriota decreased, and that of Bacteroidetes increased in the model group. At the genus level, compared with the control group, the number of Lachnospiraceae_*Clostridium, Bilophila*, and *Halomonas* were reduced in the model group, whereas the number of *Bacteroides, Parabacteroides*, and *Paraprevotella* were increased; however, nuciferine administration reversed this trend. This is in agreement with results obtained by Hua et al. (55). In addition, PCA component analysis showed that the intestinal microflora of the model group was different from that of the control group. nuciferine can improve the pathological damage induced by DSS. These data suggest that nuciferine, an alkaloid derived from the lotus leaf, can modulate the composition of the intestinal flora, and represents a promising candidate drug for the treatment of IBD.

Tight junctions connecting intestinal epithelial cells are mainly made up of claudins, ZO-1, members of the occludin family, and junctional adhesion molecules (56). Changes in the expression/cell distribution of tight junction proteins can affect the mucosal inflammatory state by affecting intramucosal homeostasis and intestinal permeability (57, 58). It is believed that high claudin expression increases intestinal permeability and promotes inflammation. In this study, we showed that the function of tight junctions was impaired after DSS treatment, and the integrity of the colorectal barrier was disrupted; however, nuciferine administration restored the function of tight junctions, protected the integrity of the intestinal barrier, and inhibited the promotion of intestinal inflammation.

## Conclusions

In conclusion, we found that nuciferine alleviates the symptoms of DSS-induced UC in mice. Nuciferine treatment reduced the DAI score, improved UC-related pathological features, restored the function of tight junctions, protected the integrity of the intestinal barrier, modulated intestinal microflora composition, and restored Treg/Th17 cell homeostasis and the Th1/Th2 balance. Combined, these results suggest that nuciferine has potential as a therapeutic compound for the treatment of UC. However, further research is needed to confirm the positive results obtained in this study.

## Data Availability Statement

The data presented in the study are deposited in the SRA database, Submission ID is SUB11619334, BioProject ID is PRJNA849750.

## Ethics Statement

The animal study was reviewed and the Legislation on the Use and Care of Laboratory Animals of the People's Republic of China and were approved by the Animal Care Review Committee of Yangtze University.

## Author Contributions

YZ: conceptualization, methodology, data curation, formal analysis, and writing-original draft. QH, YL, and JY: resources, methodology, project administration, supervision, and writing-review and editing. JL, PY, and JX: resources, validation, formal analysis, and supervision. LG: conceptualization, methodology, and writing-review and editing. GL: funding acquisition and writing-review and editing. XY and JZ: writing-review and editing. All authors contributed to the article and approved the submitted version.

## Funding

This research was funded by National Natural Science Foundation of China [Grant numbers 31602099], and Key Laboratory of Prevention and Control Agents for Animal Bacteriosis (Ministry of Agriculture) [Grant numbers KLPCAAB-2018-06].

## Conflict of Interest

The authors declare that the research was conducted in the absence of any commercial or financial relationships that could be construed as a potential conflict of interest.

## Publisher's Note

All claims expressed in this article are solely those of the authors and do not necessarily represent those of their affiliated organizations, or those of the publisher, the editors and the reviewers. Any product that may be evaluated in this article, or claim that may be made by its manufacturer, is not guaranteed or endorsed by the publisher.
